# Inflammasome Proteins Are Reliable Biomarkers of the Inflammatory Response in Aneurysmal Subarachnoid Hemorrhage

**DOI:** 10.3390/cells13161370

**Published:** 2024-08-17

**Authors:** Ruby R. Taylor, Robert W. Keane, Begoña Guardiola, Sofía López-Lage, Lesmes Moratinos, W. Dalton Dietrich, Jon Perez-Barcena, Juan Pablo de Rivero Vaccari

**Affiliations:** 1 The Miami Project to Cure Paralysis, Department of Neurological Surgery, Miller School of Medicine, University of Miami, Miami, FL 33136, USA; ruby.taylor@med.miami.edu (R.R.T.); rkeane@miami.edu (R.W.K.); ddietrich@med.miami.edu (W.D.D.); 2 Medical Scientist Training Program, Miller School of Medicine, University of Miami, Miami, FL 33136, USA; 3 Department of Cellular Physiology and Molecular Biophysics, Miller School of Medicine, University of Miami, Miami, FL 33136, USA; 4 Intensive Care Department, Son Espases University Hospital, 07120 Palma de Mallorca, Spain; maria.guardiola@ssib.es (B.G.); juan.perez@ssib.es (J.P.-B.); 5 Neurosurgical Department, Son Espases University Hospital, 07120 Palma de Mallorca, Spainlesmes.moratinos@ssib.es (L.M.)

**Keywords:** inflammasome, inflammation, aneurysm, biomarkers, cytokines, subarachnoid hemorrhage

## Abstract

Aneurysmal subarachnoid hemorrhage (aSAH) is caused by abnormal blood vessel dilation and subsequent rupture, resulting in blood pooling in the subarachnoid space. This neurological insult results in the activation of the inflammasome, a multiprotein complex that processes pro-inflammatory interleukin (IL)-1 cytokines leading to morbidity and mortality. Moreover, increases in inflammasome proteins are associated with clinical deterioration in many neurological diseases. Limited studies have investigated inflammasome protein expression following aSAH. Reliable markers of the inflammatory response associated with aSAH may allow for earlier detection of patients at risk for complications and aid in the identification of novel pharmacologic targets. Here, we investigated whether inflammasome signaling proteins may serve as potential biomarkers of the inflammatory response in aSAH. Serum and cerebrospinal fluid (CSF) from fifteen aSAH subjects and healthy age-matched controls and hydrocephalus (CSF) no-aneurysm controls were evaluated for levels of inflammasome signaling proteins and downstream pro-inflammatory cytokines. Protein measurements were carried out using Simple Plex and Single-Molecule Array (Simoa) technology. The area under the curve (AUC) was calculated using receiver operating characteristics (ROCs) to obtain information on biomarker reliability, specificity, sensitivity, cut-off points, and likelihood ratio. In addition, a Spearman *r* correlation matrix was performed to determine the correlation between inflammasome protein levels and clinical outcome measures. aSAH subjects demonstrated elevated caspase-1, apoptosis-associated speck-like protein with a caspase recruiting domain (ASC), IL-18 and IL-1β levels in serum, and CSF when compared to controls. Each of these proteins was found to be a promising biomarker of inflammation in aSAH in the CSF. In addition, ASC, caspase-1, and IL-1β were found to be promising biomarkers of inflammation in aSAH in serum. Furthermore, we found that elevated levels of inflammasome proteins in serum are useful to predict worse functional outcomes following aSAH. Thus, the determination of inflammasome protein levels in CSF and serum in aSAH may be utilized as reliable biomarkers of inflammation in aSAH and used clinically to monitor patient outcomes.

## 1. Introduction

Aneurysmal subarachnoid hemorrhage (aSAH) is caused by abnormal blood vessel dilation and subsequent rupture, resulting in blood pooling in the subarachnoid space [1,2]. Cerebral vasospasm, cortical spreading depolarization, disruption of the blood–brain barrier (BBB), and neuroinflammation contribute to morbidity and mortality following aneurysm rupture [1]. Despite advances in neurointensive care and neuroimaging technologies, approximately 35% of patients with aSAH die within 3 months, and more than 50% of survivors make an incomplete recovery [3,4]. To date, attention has been largely directed toward preventing vasospasm leading to delayed cerebral ischemia after aSAH [5,6]. However, these therapies have not translated to improved patient outcomes, indicating that other physiological processes contribute to patient well-being following aSAH [7]. 

Pathophysiological changes at the microscopic level induced by aSAH are often not visible on imaging but prove to be clinically significant in the long-term prognosis of patients. Immune responses contributing to morbidity and mortality following aSAH may be assessed using biomarkers, surrogate indicators of biological processes occurring in an individual, which have the potential to be used as predictors of outcomes after trauma [8,9]. Fluid biomarkers have been extensively studied in traumatic brain injury (TBI) and other types of neurological disease. Previous studies have shown persistently elevated serum concentrations of neuronal cell injury markers, neuron-specific enolase (NSE), and Ubiquitin C-terminal hydrolase-L1 (UCH-L1) that correlate with poor neurological outcomes and/or mortality in patients post-TBI [10,11]. Furthermore, neurofilament proteins, such as neurofilament light chain (NfL), haven proven to be important markers of neuro-axonal injury in many neurodegenerative diseases [12,13,14]. Glial cell injury markers, S100B and Glial fibrillary acidic protein (GFAP), have been shown to be predictive of TBI severity and, thus, could reduce unnecessary imaging [10,15,16,17]. However, to date, no molecular biomarker has been validated in SAH using large prospective studies.

Neuroinflammation, active in the acute and chronic phase after aSAH, contributes to unfavorable outcomes in this patient population [18,19,20]. The inflammasome is a multiprotein complex involved in the innate immune response in the central nervous system after neurological injury [21,22,23,24,25]. Inflammasomes process pro-inflammatory cytokines such as interleukin (IL)-1β and IL-18 through the activation of caspase-1. The activation of caspase-1 leads to further pro-inflammatory cytokine activation as well as the cleavage of Gasdermin-D, resulting in pyroptosis [26,27,28]. Inflammasome overactivation has been shown to play a critical role in the exacerbation of disease pathogenesis in many conditions [29,30,31]. In addition, blood breakdown products and damage-associated molecular patterns (DAMPs) released as a result of vascular and cellular compromise following aneurysm rupture can initiate the inflammatory cascade and contribute to post-aSAH complications [32,33,34]. Efforts to target inflammation following aSAH have been reported; however, limited studies have investigated inflammasome protein expression following aSAH [26,35]. Reliable markers of the inflammatory response associated with aSAH may allow for earlier detection of patients at risk for complications and aid in the identification of pharmacologic targets.

Inflammasome proteins have been previously shown to be good indicators of the inflammatory response in a variety of CNS injuries and diseases, such as traumatic brain injury [36,37], stroke [38,39,40], or Parkinson’s disease [41]. In addition, inflammasome proteins have been shown to be reliable biomarkers of the inflammation in indications outside the CNS, such as in non-alcoholic steatohepatitis [42], male pattern baldness [43], and dry eye disease. In this study, we have extended these studies to the role of inflammasome in aSAH in patients. Thus, we investigated levels of inflammasome-associated signaling proteins and the downstream pro-inflammatory cytokines IL-18 and IL-1β in the serum and CSF of aSAH subjects compared to controls. The receiver operating characteristic (ROC) curve and other biomarker characteristics were determined for each analyte tested for its potential as a biomarker of the inflammatory response associated with aSAH. Additionally, correlation matrices were performed to analyze the contribution of inflammasome signaling proteins to clinical outcomes in aSAH subjects. 

## 2. Materials and Methods

### 2.1. Participants

Serum and cerebrospinal fluid (CSF) samples from patients with aSAH and samples from non-injured control donors were used in this study. Fifteen aSAH serum and CSF samples were obtained after informed consent. Informed consent was obtained from a family member or proxy. All subjects were admitted to the Neurological Intensive Care Unit at Hospital Universitari Son Spases, Palma de Mallorca, Spain. aSAH serum and CSF samples were obtained from fifteen subjects (9 males (60%) and 6 females (40%)) with an age range of 36–77 years (median age of 64 years) (Table 1) (IRB CEI IB 4914/22). Serum samples from healthy donors were purchased from BioIVT (Hicksville, NY, USA, IRB number 20170439). The normal donor group consisted of samples obtained from 7 male and 8 female donors ages 35–68. Fifteen control CSF samples were obtained from patients with hydrocephalus admitted to Hospital Universitari Son Spases, Palma de Mallorca, Spain. aSAH samples were collected three times a day, at the same time, for the first 5 days after patients arrived at the hospital. Samples were analyzed for the 1st (Day 1), 4th (Day 2), 8th (Day 3), and 14th (Day 5) collection. Outcome measurements were performed using the Glasgow Outcome Scale Extended (GOSE) at 3 and 6 months.

### 2.2. Simple Plex Assay

Analyses of the inflammasome protein concentration in serum and CSF samples were performed using a microfluidic immunoassay, the Ella System (ProteinSimple, San Jose, CA, USA), as described in [37,41]. The Ella System was used to quantify inflammasome protein (ASC, caspase-1, and IL-18) concentrations from the serum and CSF of patients described above. 

### 2.3. Single-Molecule Array (Simoa HD-X) 

IL-1β was quantified using the Simoa IL-1β 3.0 kit in the Simoa HD-X Analyzer (Quanterix, Lexington, MA, USA) according to the manufacturer’s instructions. In brief, 185 μL of samples, calibrators, and controls were loaded into a 96 well plate and place in the respective HD-X analyzer reagent bays for the respective diluents, buffers, beads, and RGP substrates.

### 2.4. Statistical and Biomarker Analysis

Ther Simple Plex assay and Simoa HD-X data were analyzed with Prism 10 (GraphPad, Boston, MA, USA). Outliers were removed using the robust regression and outlier removal (ROUT) procedure with Q set to 1%. Normality was determined with the Shapiro–Wilk test or the D’Agostino and Pearson Test. Multiple group comparisons were carried out using a Kruskal–Wallis test ANOVA or ordinary one-way ANOVA to analyze non-parametric and parametric data, respectively. The box plot shows the minimum and the maximum with all data points. The AUC was calculated using ROC to further characterize biomarker potential and obtain information on specificity, sensitivity, cut-off points, and likelihood ratio. The accuracy was calculated, in addition to the positive and negative predictive values and the Youden index.

## 3. Results

### 3.1. aSAH Patients Have Increased CSF Levels of Inflammasome Signaling Proteins

**Figure 1:**
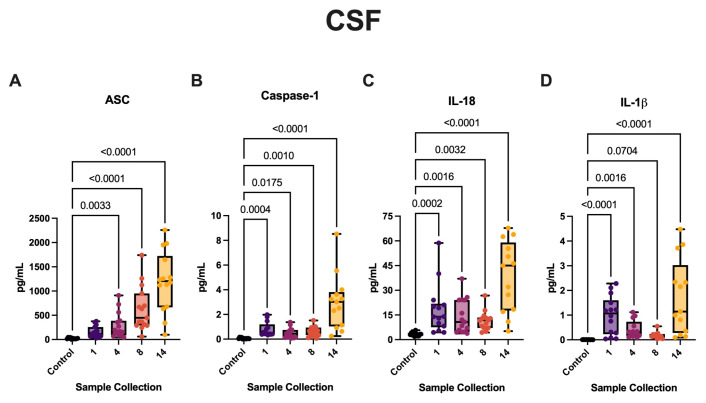
Inflammasome proteins and pro-inflammatory cytokines are increased in the CSF of aSAH subjects. The Simple Plex and Single-Molecule Array assays were used to quantify inflammasome proteins in the CSF of patients with aSAH and non-injured hydrocephalus controls. Inflammasome proteins and pro-inflammatory cytokines, (**A**) ASC, (**B**) caspase-1, (**C**) IL-18, and (**D**) IL-1β, were significantly elevated in patients with aSAH versus hydrocephalus controls. (**A**) ASC: N: control: 11, aSAH: 15; (**B**) capase-1: N: control: 12, aSAH: 13; (**C**) IL-18: N: control: 12, aSAH: 14; and (**D**) IL-1β: N: control: 9, aSAH: 14. Box and whiskers plots show the whiskers with the minimum, maximum, and all data points for each inflammatory protein of interest with respective *p*-values listed above.

Inflammasome overactivation has been previously implicated in the pathogenesis of aSAH [35,44]. To determine the biomarker potential in tissue fluids of patients with ruptured aSAH, we first evaluated the levels of inflammasome proteins in the CSF of patients with aSAH and compared them to CSF samples from patients with hydrocephalus, which were used as non-aSAH controls. Using the Ella System, we determined the levels of ASC (Figure 1A), caspase-1 (Figure 1B), and IL-18 (Figure 1C). The Simoa HD-X was used to determine the levels of IL-1β (Figure 1D). Samples were collected three times a day for 5 days. The data analyzed correspond to the 1st (day 1), 4th (day 2), 8th (day 3), and 14th (day 5) collections. aSAH patients presented significantly higher levels of caspase-1, IL-18, and IL-1β in CSF for all timepoints when compared with hydrocephalus patient controls. The levels of ASC were significantly elevated beginning at 2 days post-injury. Taken together, this indicates that inflammasome protein levels are elevated in CSF after aSAH. 

### 3.2. aSAH Patients Have Increased Serum Levels of Inflammasome Signaling Proteins

**Figure 2:**
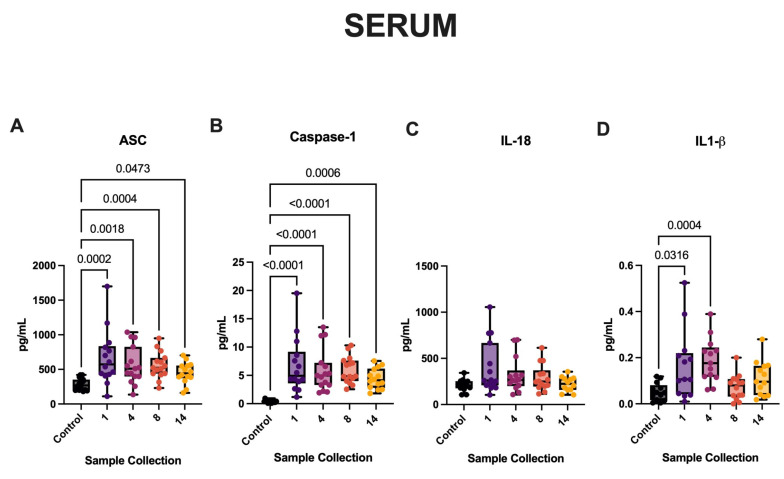
Inflammasome proteins and pro-inflammatory cytokines are increased in the serum of aSAH subjects. The Simple Plex and Single-Molecule Array assays were used to quantify inflammasome proteins in the serum of patients with aSAH and healthy, age-matched controls. (**A**) ASC, and (**B**) caspase-1 were significantly elevated at all timepoints versus healthy, age-matched controls. (**D**) IL-1β was found to be significantly elevated up to two days post-injury. (**C**) IL-18 was not significantly elevated in aSAH patients. (**A**) ASC: N: control: 15, aSAH: 14; (**B**) capase-1: N: control: 14, aSAH: 14; (**C**) IL-18: N: control: 14, aSAH: 15; and (**D**) IL-1β: N: control: 13, aSAH: 13. Box and whiskers plots show the whiskers with the minimum, maximum, and all data points for each inflammatory protein of interest with respective *p*-values listed above.

Since CSF collection implies an invasive procedure, we aimed to determine whether increased inflammasome protein expression was also evident in the serum of this patient cohort at the same timepoints examined above. aSAH patients had significantly higher levels of ASC (Figure 2A) and caspase-1 (Figure 2B) up to 5 days post-injury when compared to healthy controls. However, the level of IL-1β (Figure 2D) was significantly elevated only up to 2 days post-injury in the serum of patients with aSAH when compared with healthy controls. aSAH patients did not demonstrate significantly higher levels of IL-18 (Figure 2C) when compared with serum controls. 

### 3.3. Inflammasome Proteins in the CSF Are Reliable Biomarkers of aSAH 

**Figure 3:**
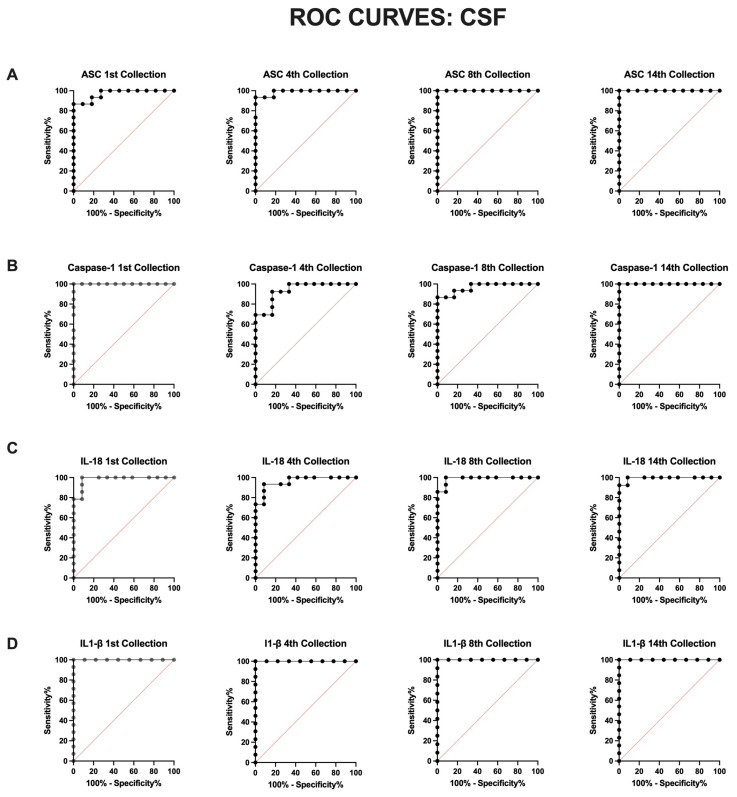
ROC for inflammatory biomarkers of aSAH in CSF. The ROC and AUC were calculated for (**A**) ASC, (**B**) caspase-1, (**C**) IL-18, and (**D**) IL-1β as they were previously identified to be significantly elevated in the CSF of patients with aSAH when compared to hydrocephalus controls. (**A**) ASC: N: control: 11, aSAH: 15; (**B**) capase-1: N: control: 12, aSAH: 13; (**C**) IL-18: N: control: 12, aSAH: 14; and (**D**) IL-1β: N: control: 9, aSAH: 14.

To determine whether inflammasome proteins are reliable biomarkers of aSAH in CSF and serum, we plotted the receiver operating characteristic (ROC) curve for each protein and determined the area under the curve (AUC) for each timepoint (Table 2). Accordingly, in the CSF, IL-1β (Figure 3D) had the highest AUC values for all timepoints at 1.0 (Table 2) with a specificity and sensitivity of 100% (Table 3). Caspase-1 (Figure 3B) also had an AUC of 1.0 (Table 2) with a specificity and sensitivity of 100% (Table 3) for the 1st and 14th collection. ASC (Figure 3A) also presented an AUC of 1.0 (Table 2) for the 8th and 14th collection with a specificity and sensitivity of 100% (Table 3), whereas IL-18 presented a high AUC value (0.99) (Table 2) at the 14th collection with a sensitivity of 100% and specificity of 91.67% with a cut-off point of >4.65 pg/mL. Overall, the results indicate that all four inflammatory proteins are reliable CSF biomarkers of the inflammatory response following aSAH up to 5 days post-injury. 

#### 3.3.1. Inflammasome Proteins in Serum Are Reliable Biomarkers of aSAH 

**Figure 4:**
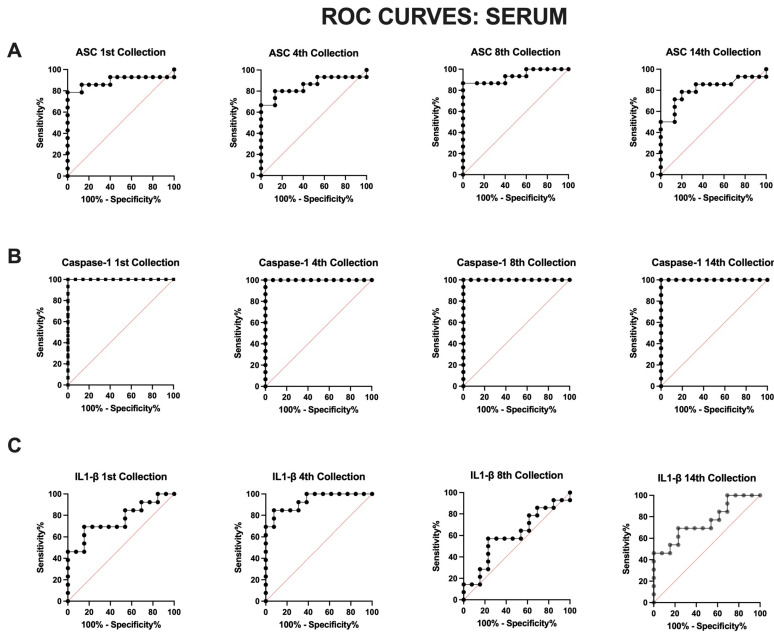
ROC for inflammatory biomarkers of aSAH in serum. The ROC and AUC were calculated for (**A**) ASC, (**B**) caspase-1, and (**C**) IL-1β as they were previously identified to be significantly elevated in serum of patients with aSAH when compared to healthy, age-matched controls. (**A**) ASC: N: control: 15, aSAH: 14; (**B**) capase-1: N: control: 14, aSAH: 14; and (**C**) IL-1β: N: control: 13, aSAH: 13.

In serum, caspase-1 (Figure 4B) had the highest AUC value (1.0) (Table 4) for all timepoints with a sensitivity and specificity of 100% (Table 5). ASC (Figure 4A) also demonstrated a high AUC value of 0.93 (Table 4) with a sensitivity of 100% and specificity of 40% for the eighth collection with a cut-off point of 224 pg/mL (Table 5). IL-1β (Figure 4C) presented a high AUC value of 0.94 (Table 4) with a sensitivity of 100% and specificity of 61.54% for the fourth collection with a cut-off point of >0.06 (Table 5). These results demonstrate that caspase-1, ASC, and IL-1β are reliable serum biomarkers of the inflammatory response following aSAH up to 5 days post-injury.

#### 3.3.2. Correlation between Inflammasome Biomarkers in CSF and Serum 

**Figure 5:**
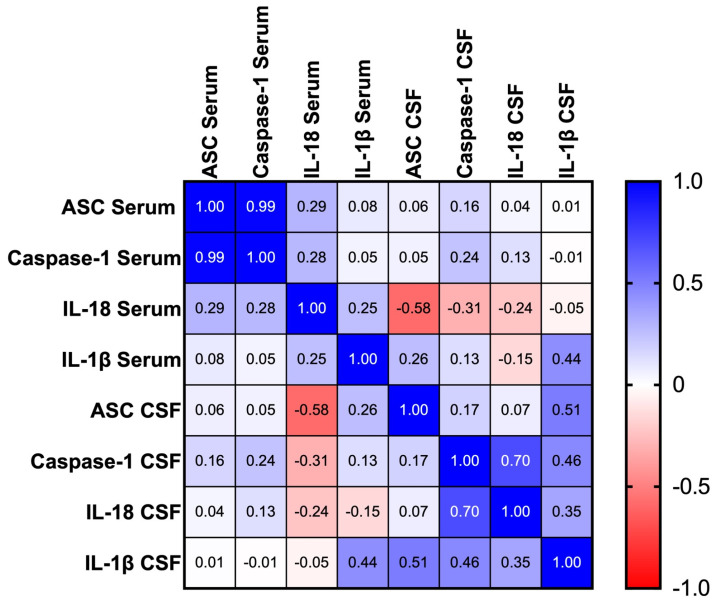
Correlation matrix among inflammatory biomarkers in aSAH. Correlation matrix using a Spearman correlation among biomarkers. *p*-values of significance (<0.05) for comparison of ASC vs. caspase-1 in serum. *p*-values of significance (<0.05) for the following comparison of CSF biomarkers: caspase-1 vs. IL-18 and ASC vs. IL-1β. *p*-values of significance (<0.05) for the following comparison of serum vs. CSF biomarkers: IL-18 in serum vs. ASC in CSF.

To determine the correlation across serum and CSF levels of ASC, caspase-1, IL-18, and IL-1β in aSAH patients, a Spearman *r* correlation matrix was performed (Figure 5). In the serum, the greatest positive correlation was found between ASC and caspase-1 with an *r* = 0.99 (*p* < 0.001). 

In the CSF, the greatest positive correlation was found between caspase-1 and IL-18 with an *r* = 0.70 (*p* = 0.009). There was also a high positive correlation between ASC and IL-1β with an *r* = 0.51 (*p* = 0.064). Interestingly, between the serum and CSF, there was a negative correlation between IL-18 in the serum and ASC in the CSF with an *r* = −0.58 (*p* = 0.025). 

#### 3.3.3. Correlation between Inflammasome Biomarkers and Clinical Outcomes 

**Figure 6:**
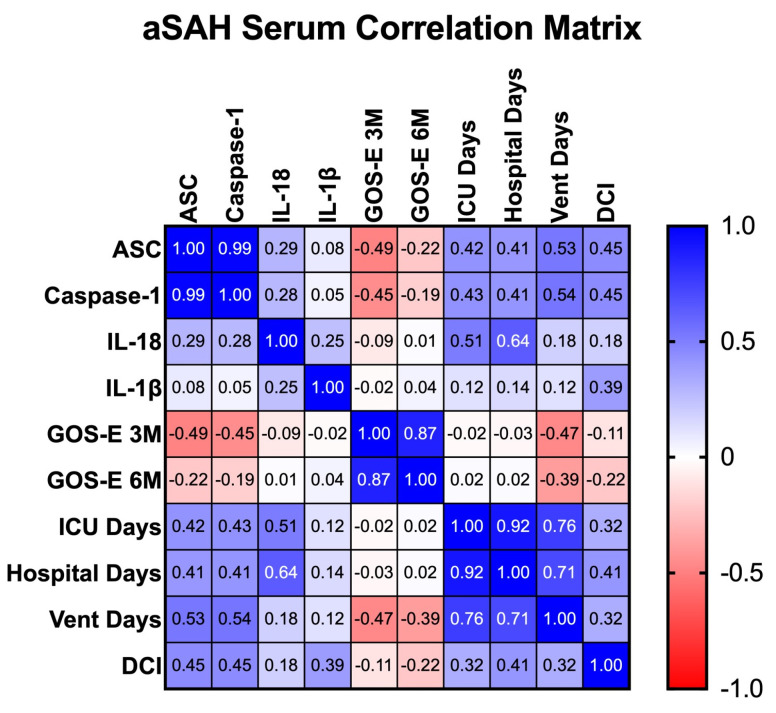
Correlation matrix between serum inflammatory biomarkers and clinical outcomes in aSAH. Correlation matrix using a Spearman correlation between serum biomarkers and clinical outcome measures. *p*-values of significance (<0.05) for the following comparisons of serum biomarkers versus clinical outcome measures: caspase-1 vs. vent days; IL-18 vs. hospital days. DCI = Delayed Cerebral Ischemia, GOS-E 3M = Glasgow Outcome Scale-Extended 3-Months Post Injury, GOS-E 6M = Glasgow Outcome Scale-Extended 6-Months Post Injury.

To determine the correlation across serum levels of inflammatory proteins for the first collection point (1 day post-injury) and clinical outcome measures, a Spearman *r* correlation matrix was performed (Figure 6). The strongest correlation was between IL-18 and number of days spent in hospital with an *r* = 0.64 (*p* = 0.012). We also found a high correlation between ASC and caspase-1 and the number of days a patient was on mechanical ventilation with an *r* = 0.53 (*p* = 0.054) and an *r* = 0.54 (*p* = 0.049), respectively. Together, these findings suggest that high levels of inflammasome proteins may serve as an indicator of poor outcomes following aSAH. This identical approach was used to determine the correlation across CSF levels of inflammatory proteins and clinical outcome measures. However, we did not find strong correlations between CSF levels of inflammasome proteins and clinical outcome measures (Figure S1).

## 4. Discussion

aSAH is a multifactorial condition often resulting in systemic organ dysfunction [45]. Despite significant advances in neurocritical care, patient outcomes remain poor with a 45% mortality rate within 30 days and 50% of surviving patients suffering from irreversible brain damage [46]. Factors influencing prognosis include pre-aSAH health status (e.g., hypertension, old age, and history of vascular disease), SAH severity, and post-aSAH complications [47,48]. The inflammatory response following aSAH may mediate post-aSAH complications. With aneurysm rupture, blood pours into the subarachnoid space, triggering a cascade of inflammatory responses. As a result, immune cells are activated, leading to the release of chemokines and secretion of pro-inflammatory cytokines, thus further exacerbating inflammation [49]. Excessive inflammation exacerbates brain edema, BBB disruption, and neuronal apoptosis, which leads to further clinical deterioration in patients with aSAH [49,50]. In addition, the reduction of inflammation-mediated processes might have great therapeutic potential to reduce post-aSAH complications. Despite previous studies demonstrating the role of cellular and molecular inflammation in aSAH disease pathogenesis, current therapeutics fail to mitigate neuroinflammation following aneurysm rupture [47]. Hence, a deeper understanding of the role of the inflammatory response following aSAH should allow improvements in diagnostic accuracy, guide management, and prevent long-term complications. 

In this proof-of-concept study, we used two advanced technologies, microfluidics and Single-Molecule Array, to measure inflammasome signaling proteins and downstream pro-inflammatory cytokines in the serum and CSF of 15 patients with aSAH. aSAH patient samples were collected upon admission and daily up to 5 days post-injury. In this study, we chose acute timepoints consistent with the acute nature of the innate immune response. Moreover, sample timepoints were chosen based off the existing literature of other neurological injuries in which inflammasome protein expression peaks between 24 and 5 days following injury [51,52]. Furthermore, while a more chronic timepoint would be interesting, a previous study found that the presence of infection and an elevated temperature at day 10 were associated with the development of delayed cerebral ischemia (DCI), a common complication of aSAH [53]. However, the serum concentration of inflammatory markers was not associated with DCI in this cohort [53]. Furthermore, inflammatory markers are often elevated in chronic timepoints due to the nature of the patient being in the Intensive Care Unit and complications such as ventilator-associated infections and urinary tract infections. In addition, the clinical utility of a biomarker increases when an acute sample can be predictive of long-term outcome. aSAH patient serum and CSF were compared to healthy, age-matched controls and hydrocephalus non-aSAH patient controls, respectively. The etiology of hydrocephalus in the CSF patient control group was heterogenous with diagnoses including adult-onset chronic hydrocephalus, idiopathic intracranial hypertension, obstructive hydrocephalus, communicating hydrocephalus, and posthemorrhagic hydrocephalus (Table S1). In addition, due to the difficult and invasive nature of CSF collection, a limitation of the study is the variance in time of CSF collection. Despite the heterogenous control group, we demonstrate that aSAH patients had significantly higher levels of ASC, caspase-1, IL-18, and IL-1β up to 5 days post-injury when compared to hydrocephalus non-aSAH patient controls. Furthermore, inflammasome signaling proteins demonstrated robust specificity, sensitivity, and AUC values above 0.9, indicating they are potential reliable biomarkers of the inflammatory response in aSAH. We also identified significant increases in ASC and caspase-1 up to 5 days-post injury in serum when compared with age-matched controls. In addition, the level of inflammasome protein IL-1β was significantly elevated up 2 days post-injury. Additionally, using correlation matrices, we observed significant correlations between inflammasome proteins in the serum and clinical outcome measures in patients with aSAH, suggesting inflammasome overactivation could contribute to poor clinical outcomes in this patient population. 

Biomarkers, surrogate indicators of pathological or normal processes, may aid in the identification of prognostic factors and better inform clinical practice. In particular, serum and CSF markers may be advantageous as pathophysiological changes induced by neurological injury are often not visible on CT or MRI imaging but prove to be significant in long-term prognosis [54]. In addition, fluid markers avoid radiation exposure induced by CT and are more cost-effective. CSF, peripheral blood, and cerebral blood have been investigated as sources for the detection of biomarkers [47,55]. The most studied biomarkers of inflammation include tumor necrosis factor (TNF), C-reactive protein (CRP), tissue factor (TF), and matrix metalloproteinase-9 (MMP-9). TNF mediates many inflammatory processes, has been found to be elevated in the brain parenchyma, serum, and CSF 2–10 days following aSAH, and may exacerbate BBB disruption and neuronal apoptosis [47,56]. CRP has also been investigated as a serum biomarker following aSAH. Patients with low GCS and high Hunt–Hess and Fisher grades have been shown to present with elevated serum CRP levels [57,58]. Furthermore, angiographic vasospasm, a known complication of aSAH, has been correlated with elevated serum and CSF CRP levels [57]. In a recent study, tissue factor (TF) serum levels were found to positively correlate with aSAH severity [35]. Finally, MMP-9 is produced in many inflammatory cells and has a role in aggravating tissue damage via the upregulation of chemokines [47]. Previous studies have demonstrated that elevated MMP-9 levels are correlated with higher delayed cerebral ischemia rates, a common complication of aSAH, and worse 3-month functional outcomes [47]. 

Inflammasomes are large protein complexes that form in response to infectious agents or cellular damage [59]. The inflammasome controls sterile inflammation pathways and has been investigated in the exacerbation of many diseases due to its role in post-translational processing of pro-inflammatory cytokines [60]. In the presence of damage-associated molecular patterns (DAMPs) and other stimuli, inflammasome sensors promote assembly by recruiting ASC [59]. After the recruitment of ASC, autoproteolytic cleavage of caspase-1 leads to the formation of mature caspase-1 [59]. Once active, caspase-1 processes pro-IL-1β and IL-18 to induce the secretion of IL-1β and IL-18. In addition, caspase-1 is capable of cleaving Gasdermin-D to trigger the program cell death process of pyroptosis [59,61,62]. Many inflammasome sensors have been identified, but only a few have been characterized in central nervous system diseases. In the setting of aneurysm rupture, erythrocyte lysis and the release of soluble factors into the bloodstream compounded with BBB disruption can promote NLR family pyrin domain-containing 3 (NLRP3) inflammasome activation [35,63]. A previous study showed that NLRP3 inhibition with MCC950 prevented middle cerebral artery vasospasm and decreased SAH-induced sensorimotor defects in a mouse model of SAH [61]. Furthermore, a recent study demonstrated increased levels of NLRP3, ASC, and caspase-1 in monocytes from patients with aSAH when compared to controls [35]. While our study focused on the NLRP3 inflammasome, absent in melanoma 2 (AIM2) induces a specific type inflammasome in neurons [27] that leads to pyroptosis [64]. A previous study investigated the role of the AIM2 inflammasome in early brain injury in patients with aSAH [27]. In that study, the authors concluded that the level of AIM2 in the CSF of patients with aSAH was significantly higher than in control patients and elevated levels of AIM2 in CSF were correlated with a higher Hunt–Hess grade [27].

Despite establishing inflammasome overactivation in patients with aSAH, few studies have examined the biomarker potential of these signaling proteins for prognostic purposes. Importantly, to perform a comprehensive biomarker study, it is necessary to determine the ROC curve and to obtain the AUC value to determine biomarker reliability [5]. Here, we obtained ROC curves plotting biomarker sensitivity and specificity, as well as cut-off points that maximized sensitivity and specificity. According to our analyses, the pro-inflammatory cytokine IL-1β was found to be a reliable biomarker in CSF with an AUC of 1.00 at all timepoints and a specificity and sensitivity of 100%. Moreover, the inflammasome signaling proteins, caspase-1 and ASC, were also reliable biomarkers in CSF with an AUC of 1.00 at the 1st and 14th collection and 8th and 14th collection, respectively. These biomarkers also presented with high specificity and sensitivity. IL-18 also demonstrated a high AUC value greater than 0.90 for the 14th collection with robust sensitivity and specificity. Overall, the results indicate that all four proteins are reliable CSF biomarkers of the inflammatory response following aSAH. In serum, caspase-1 had the highest AUC value (1.00) for all timepoints examined with a sensitivity and specificity of 100%. Therefore, caspase-1 is the most reliable serum biomarker of the inflammatory response following aSAH of all the analytes tested. ASC and IL-1β also demonstrated an AUC greater than 0.90 with a sensitivity and specificity greater than 80% for the eighth and fourth collection, respectively, and they are, therefore, reliable serum biomarkers of the inflammatory response following aSAH primarily at 2 days post-injury. While the biomarkers analyzed were reliable at the analyzed timepoints, it is important to note that in a healthy state, inflammasome protein expression and downstream cytokine expression are under circadian transcriptional control [65]. However, this temporal regulation is disrupted in injured, inflammatory diseased states. In our study, we analyzed the first sample from day 1 and 2 post-injury and the second sample taken on day 3 and 5 post-injury. Future studies will investigate the temporal profile of inflammasome protein expression following aSAH. By assessing serum and CSF biomarkers, we can analyze the inflammatory response associated with aSAH on a local and systemic level. The establishment of inflammatory proteins as blood plasma biomarkers can have substantial clinical potential to provide prognostic information in a less invasive procedure.

We also investigated whether inflammasome proteins are correlated with clinical outcomes in patients with aSAH and found a strong correlation between serum levels of IL-18 and the number of days patients were hospitalized with an *r* = 0.64. We also found a high correlation between serum levels of ASC and caspase-1 and the number of days patients were on mechanical ventilation with an *r* = 0.53 and an *r* = 0.54, respectively. These findings suggest that high levels of inflammasome proteins in serum can serve as an indicator of poor outcome following aSAH. Interestingly, we did not find any strong correlations between CSF levels of inflammasome proteins and clinical outcome measures. In this study, we found no correlation between CSF biomarkers and outcomes, but we did see it in serum. It is possible that the lack of correlation between CNS biomarkers in the CSF and outcomes is potentially due to the instability of proteins in the CSF. This has been previously reported in other fields such as Alzheimer’s disease [66]. Furthermore, we hypothesize the correlation with outcomes in serum could be due to the numerous systemic complications of aneurysmal subarachnoid hemorrhage. For example, common pulmonary complications of aSAH include neurogenic pulmonary edema and pulmonary embolism [67]. Previous studies of traumatic brain injury have demonstrated the inflammasome has an important role in the systemic inflammatory response leading to lung injury following TBI [68]. In addition, EKG abnormalities occur in almost 100% of patients with aSAH [67]. TBI has been shown to elicit inflammasome activation in the atrium, further contributing to systemic inflammation following injury [21]. However, organ-specific inflammasome activation in the context of aSAH has yet to be elucidated. Aside from systemic complications, this difference could be attributed to the time difference between inflammasome protein expression in serum and CSF. Future studies should carry out CSF collection longer than 5 days to elucidate whether CSF proteins correlate with clinical outcome measures such as delayed cerebral ischemia, vasospasm, or cerebral infarcts in the CT or MRI, as well as other clinical outcome measures in more chronic stages, as we have previously shown in patients after traumatic brain injury [37]. Furthermore, longer clinical observation and the inclusion of patient follow-up data could increase the strength of correlations of biomarker and clinical findings. 

This proof-of-concept study reveals that inflammasome signaling proteins and downstream pro-inflammatory cytokines are reliable biomarkers of inflammatory events associated with aSAH. The identification of the inflammasome signaling proteins after aSSH may help develop therapeutics to dampen the exacerbated inflammation after aSAH and to obtain prognostic information to monitor patient outcomes. 

## Figures and Tables

**Table 1:** Patient characteristics of subjects with aneurysm rupture.

Sex	Males	9 (60%)
	Females	6 (40%)
Age	Range	36–77
	Median	64
GCS	Average	9
	GCS 3	2 (13%)
	GCS 4	2 (13%)
	GCS 5	1 (6%)
	GCS 6	1 (6%)
	GCS 8	1 (6%)
	GCS 9	1 (6%)
	GCS 12	1 (6%)
	GCS 13	2 (13%)
	GCS 14	2 (13%)
	GCS 15	2 (13%)
WFNS scale	Average	3
	WFNS 1	2 (13%)
	WFNS 2	4 (27%)
	WFNS 4	4 (27%)
	WFNS 5	5 (33%)
Fisher scale	Average	4
	Fisher 3	1 (6%)
	Fisher 4	14 (94%)
ICU Days	Range	17–90
	Median	23
Hospital Days	Range	16–126
	Median	32

GCS: Glasgow Coma Scale, WFNS: World Federation Neurological Scale, ICU: Intensive Care Unit.

**Table 2:** ROC analysis results for CSF proteins including area under the curve, standard error (STD Error), 95% confidence interval (CI), and *p*-value for 1st, 4th, 8th, and 14th collections.

CSF Collection	Area	STD Error	95% C.I.	*p*-Value
ASC				
1st Collection	0.969	0.028	0.915 to 1.000	<0.0001
4th Collection	0.988	0.016	0.957 to 1.000	<0.0001
8th Collection	1.000	0.000	1.000 to 1.000	<0.0001
14th Collection	1.000	0.000	1.000 to 1.000	<0.0001
Caspase-1				
1st Collection	1.000	0.000	1.000 to 1.000	<0.0001
4th Collection	0.936	0.046	0.846 to 1.000	0.0002
8th Collection	0.967	0.029	0.909 to 1.000	<0.0001
14th Collection	1.000	0.000	1.000 to 1.000	<0.0001
IL-18				
1st Collection	0.982	0.021	0.941 to 1.000	<0.0001
4th Collection	0.961	0.033	0.897 to 1.000	<0.0001
8th Collection	0.988	0.016	0.957 to 1.000	<0.0001
14th Collection	0.994	0.011	0.913 to 1.000	<0.0001
IL-1β				
1st Collection	1.000	0.000	1.000 to 1.000	<0.0001
4th Collection	1.000	0.000	1.000 to 1.000	<0.0001
8th Collection	1.000	0.000	1.000 to 1.000	0.0001
14th Collection	1.000	0.000	1.000 to 1.000	<0.0001

**Table 3:** ROC analysis results for CSF proteins including cut-off point, sensitivity, specificity, Youden index, likelihood ratio (LR), positive predictive value (PPV), negative predictive value (NPV), and accuracy for 1st, 4th, 8th, and 14th collections.

CSF Collection	Cut-Off Point (pg/mL)	Sensitivity (%)	Specificity (%)	Youden Index	LR	PPV	NPV	Accuracy (%)
ASC								
1st Collection	>25.85	100	72.73	0.73	3.67	83	100	88
4th Collection	>27.20	100	72.73	0.73	3.67	83	100	88
8th Collection	>52.65	100	100	1		100	100	100
14th Collection	>71.95	100	100	1		100	100	100
Caspase-1								
1st Collection	>0.24	100	100	1		100	100	100
4th Collection	>0.08	92.31	83.33	0.76	5.54	86	91	88
8th Collection	>0.06	100	66.67	0.67	3.00	79	100	85
14th Collection	>0.20	100	100	1		100	100	100
IL-18								
1st Collection	>4.29	100	91.67	0.92	12.00	93	100	96
4th Collection	>4.31	93	91.67	0.85	11.20	93	92	93
8th Collection	>4.27	100	91.67	0.92	12.00	93	100	96
14th Collection	>4.65	100	91.67	0.92	12.00	93	100	96
IL-1β								
1st Collection	>0.02	100	100	1		100	100	100
4th Collection	>0.05	100	100	1		100	100	100
8th Collection	>0.02	100	100	1		100	100	100
14th Collection	>0.05	100	100	1		100	100	100

**Table 4:** ROC analysis results for serum proteins including area under the curve, standard error (STD Error), 95% confidence interval (CI) and *p*-value for 1st, 4th, 8th, and 14th collections.

Serum Collection	Area	STD Error	95% C.I.	*p*-Value
ASC				
1st Collection	0.890	0.074	0.746 to 1.000	0.0003
4th Collection	0.853	0.076	0.703 to 1.000	0.0010
8th Collection	0.933	0.048	0.837 to 1.000	<0.0001
14th Collection	0.818	0.087	0.641 to 0.982	0.0043
Caspase-1				
1st Collection	1.000	0.000	1.000 to 1.000	<0.0001
4th Collection	1.000	0.000	1.000 to 1.000	<0.0001
8th Collection	1.000	0.000	1.000 to 1.000	<0.0001
14th Collection	1.000	0.000	1.000 to 1.000	<0.0001
IL-1β				
1st Collection	0.763	0.096	0.575 to 0.952	0.0225
4th Collection	0.935	0.046	0.846 to 1.000	0.0002
8th Collection	0.604	0.112	0.386 to 0.8.23	0.3565
14th Collection	0.757	0.956	0.569 to 0.945	0.0257

**Table 5:** ROC analysis results for serum proteins including cut-off point, sensitivity, specificity, Youden index, likelihood ratio (LR), positive predictive value (PPV), negative predictive value (NPV), and accuracy for collections 1st, 4th, 8th, and 14th.

Serum Collection	Cut-Off Point (pg/mL)	Sensitivity (%)	Specificity (%)	Youden Index	LR	PPV	NPV	Accuracy (%)
ASC								
1st Collection	>296	92.86	60.00	0.53	2.321	70	89	76
4th Collection	>255.5	93.33	46.67	0.40	1.750	64	87	70
8th Collection	>224	100	40.00	0.40	1.667	100	95	70
14th Collection	>331	85.71	66.67	0.52	2.571	72	82	76
Caspase-1								
1st Collection	>1.052	100	100	1		100	100	100
4th Collection	>1.432	100	100	1		100	100	100
8th Collection	>1.747	100	100	1		100	100	100
14th Collection	>1.377	100	100	1		100	100	100
IL-1β								
1st Collection	>0.038	84.62	46.15	0.31	1.571	61	75	65
4th Collection	>0.055	100	61.54	0.62	2.600	72	100	81
8th Collection	> 0.027	78.57	30.77	0.99	1.135	57	53	56
14th Collection	>0.0248	92.31	30.77	0.23	1.333	57	80	62

## Data Availability

Available data will be provided upon request to the corresponding author.

## Supplementary Materials

The following supporting information can be downloaded at: https://www.mdpi.com/article/10.3390/cells13161370/s1, Figure S1: Correlation between inflammasome proteins and clinical outcomes in CSF. Table S1: CSF control patient characteristics.
