# Supplementary material for: Inflammasome Proteins Are Reliable Biomarkers of the Inflammatory Response in Aneurysmal Subarachnoid Hemorrhage
Source: Cells. 2024 Aug 17;13(16):1370. doi: 10.3390/cells13161370 (PMC11353247; doi:10.3390/cells13161370)
Supplement: Supplementary file 1 [file cells-13-01370-s001.zip › cells-3114115-supplementary.pdf]

Figure S1.

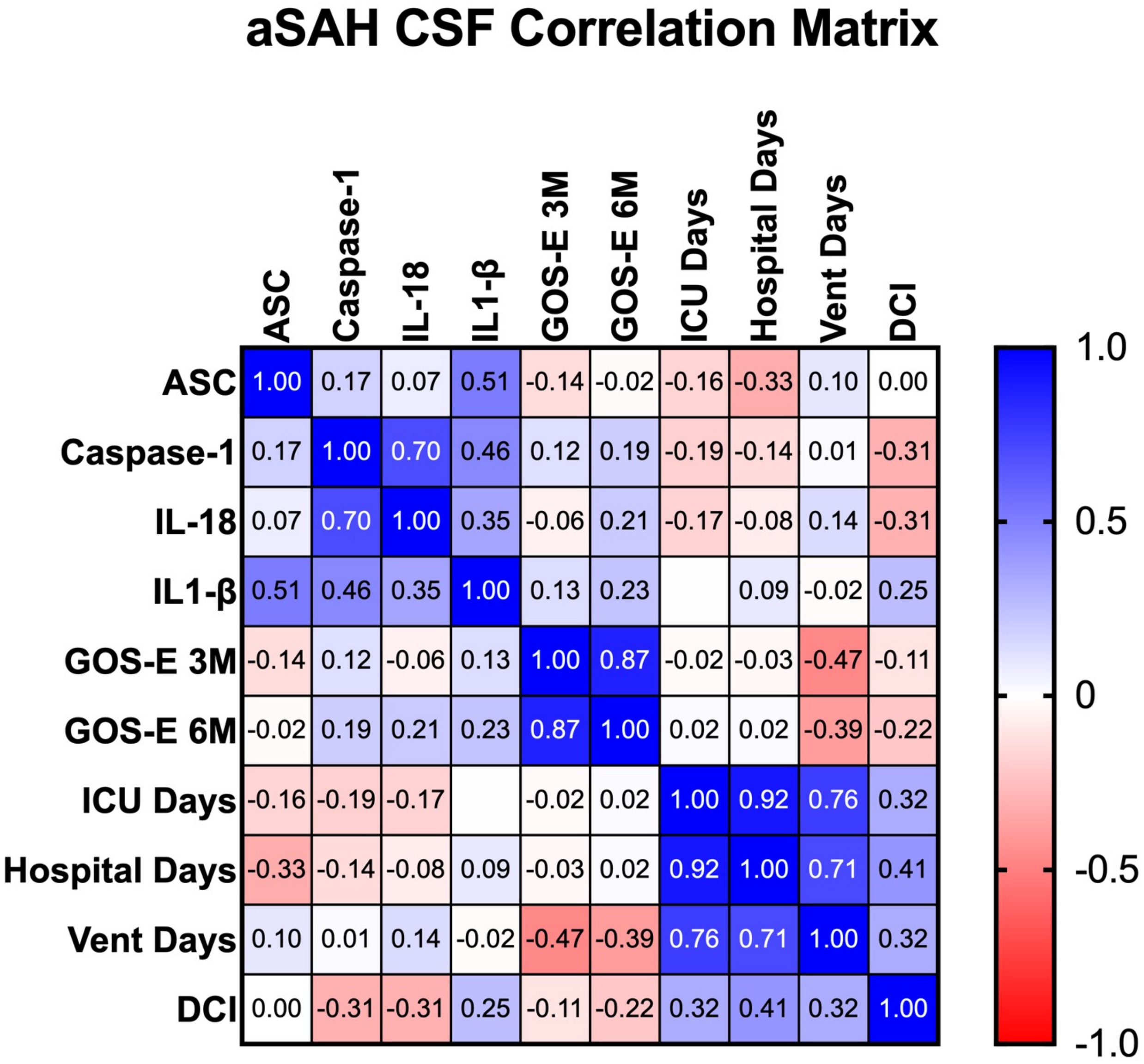

Table S1. CSF Control Patient Characteristics

Sex	Males	6 (40%)
	Females	9 (60%)
Age	Range	17 - 79
	Median	56
Diagnosis	Adult-Onset Chronic Hydrocephalus	8 (53%)
	Idiopathic Intracranial Hypertension	1 (6%)
	Obstructive Hydrocephalus	3 (20%)
	Communicating Hydrocephalus	2 (13%)
	Posthemorrhagic Hydrocephalus	1 (6%)
